# The impact of stem fixation method on Vancouver Type B1 periprosthetic femoral fracture management

**DOI:** 10.1051/sicotj/2021064

**Published:** 2022-01-06

**Authors:** Katherine Wang, Eustathios Kenanidis, Zakareya Gamie, Khurram Suleman, Mark Miodownik, Mahsa Avadi, David Horne, Jonathan Thompson, Eleftherios Tsiridis, Mehran Moazen

**Affiliations:** 1 Department of Mechanical Engineering, University College London Torrington Place London WC1E 7JE UK; 2 Academic Orthopaedics Department, Papageorgiou General Hospital & CORE Lab at CIRI AUTH, Aristotle University Medical School, University Campus 54 124 Thessaloniki Greece; 3 Northern Institute for Cancer Research, Newcastle University Framlington Place Newcastle upon Tyne NE2 4HH UK; 4 DePuy Synthes St. Anthony’s Road Leeds LS11 8DT UK

**Keywords:** Total hip arthroplasty (THA), Periprosthetic femoral fracture (PFF), Biomechanics, Cemented, Uncemented

## Abstract

*Introduction:* Our understanding of the impact of the stem fixation method in total hip arthroplasty (THA) on the subsequent management of periprosthetic femoral fractures (PFF) is still limited. This study aimed to investigate and quantify the effect of the stem fixation method, i.e., cemented vs. uncemented THA, on the management of Vancouver Type B1 periprosthetic femoral fractures with the same plate. *Methods:* Eight laboratory models of synthetic femora were divided into two groups and implanted with either a cemented or uncemented hip prosthesis. The overall stiffness and strain distribution were measured under an anatomical one-legged stance. All eight specimens underwent an osteotomy to simulate Vancouver type B1 PFF’s. Fractures were then fixed using the same extramedullary plate and screws. The same measurements and fracture movement were taken under the same loading conditions. *Results:* Highlighted that the uncemented THA and PFF fixation constructs had a lower overall stiffness. Subsequently, the mechanical strain on the fracture plate for the uncemented construct was higher compared to the cemented constructs. *Conclusion:* PFF fixation of a Vancouver type B1 fracture using a plate may have a higher risk of failure in uncemented THAs.

## Introduction

Periprosthetic femoral fractures (PFF) occur following total hip arthroplasty (THA) in various fracture configurations at different locations. Treatment of these can be challenging and complex and is determined by multiple factors such as bone quality, fracture topography, and stem type [1–5]. The Vancouver classification system is the most widely used classification system for PFF [6, 7], with Type B fractures accounting for the greatest number of cases (ca. 70%). This system considers fracture level and bone quality. Still, it does not consider whether the stem is cemented or uncemented and thus does not distinguish whether there is a difference in treatment between the two.

To the best of our knowledge, despite the large clinical literature comparing cemented vs. uncemented THAs (e.g., [2]), there are few biomechanical studies between the two [8–13]. These studies have typically (1) investigated the risk of periprosthetic fracture associated with different types of THAs, and the features of the stem design (2) highlighted that cemented constructs have a higher overall stiffness compared to the uncemented constructs [8, 9]. If this is the case, then one can expect that the PFF fixation of the two constructs should be treated differently to reduce the risk of failure for the uncemented constructs. However, there is no biomechanical study comparing PFF fixation of cemented vs. uncemented THAs [14, 15]. Hence the rationale behind this study was to investigate if there is any difference between the PFF fixation of the cemented and uncemented THAs. If so, would the fixation of the uncemented constructs undertake a higher level of mechanical strain (i.e., being at a higher risk of failure) or not.

Hence, the aim of this study was to investigate and quantify the effect of the stem implantation method, i.e., cemented vs. uncemented THA, on the management of Vancouver Type B1 periprosthetic femoral fractures with the same plate fixation method.

## Methods

Cemented and uncemented total hip arthroplasties were performed on surrogate femurs. Following measurement of the constructs’ overall performance (stiffness) and the level of mechanical strain in several areas, periprosthetic femoral fracture fixation was replicated by fracturing the THA constructs and fixing them using extramedullary plate and screws. The overall performance of the PFF fixation constructs and the pattern of the mechanical strain across the plates and fracture movements were measured to understand the impact of the two considered THAs on the plate fixation.

### Specimens

**Figure 1:**
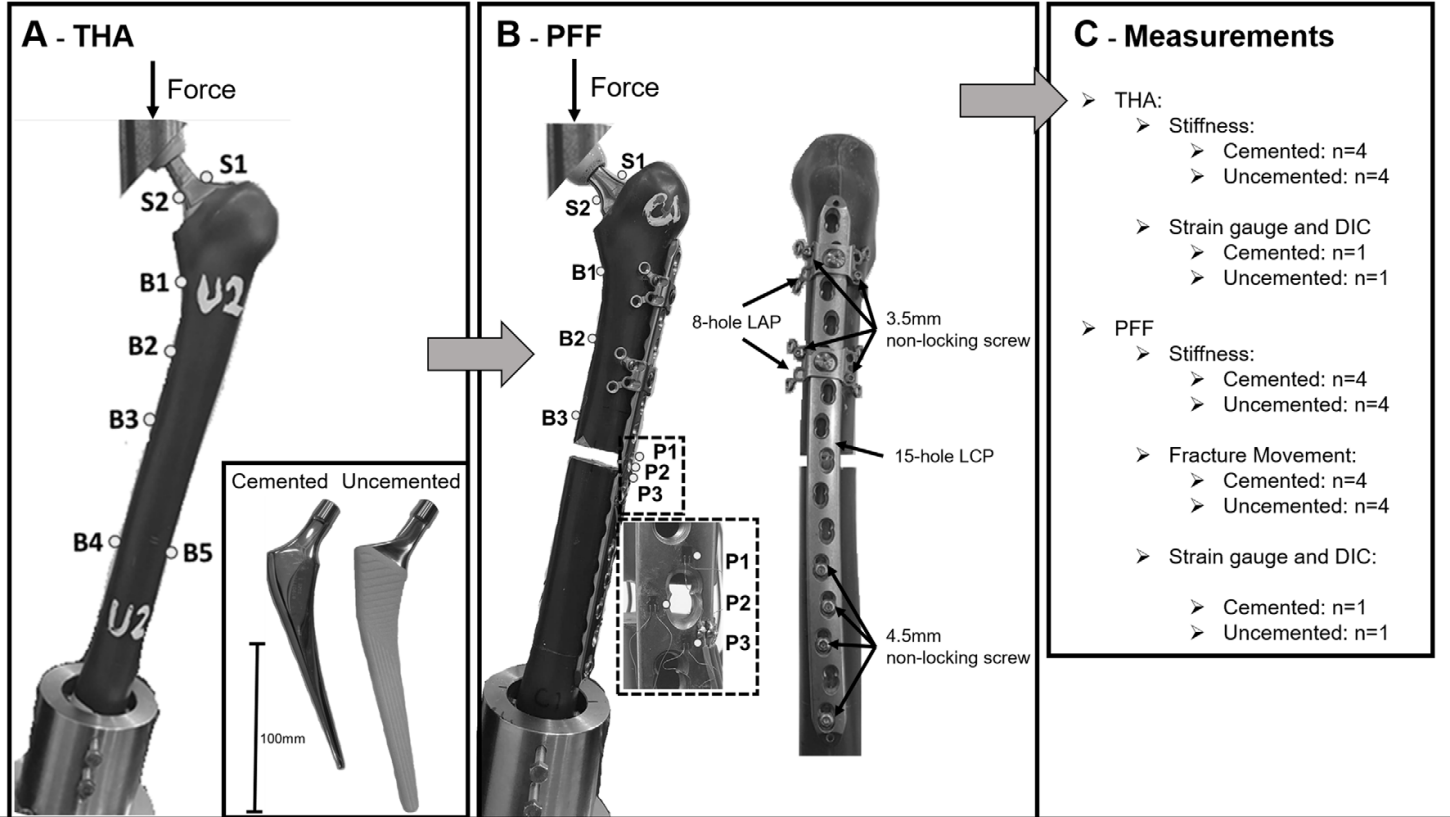
Overview of the study. Experimental setup of total hip replacement (A) and periprosthetic femoral fracture fixation (B). S1–S2 highlight strain gauge attachment site on the stem, B1–B5 highlight strain gauge attachment site on the bone, and P1–P3 highlight strain gauge attachment site on the fracture plate.

Eight large left, fourth-generation composite femurs (Sawbones Worldwide, WA) were used in this study. The femora were randomized into two groups with four constructs each, to first simulate cemented and uncemented THAs and then simulate cemented and uncemented Vancouver type B1 PFFs – with the fracture located around the stem with a stable implant and good bone quality [6] (Figure 1).

Specimens were prepared by removing the femoral condyles, 60 mm from the distal femur. Cemented THA was performed using the DePuy C-Stem® AMT standard offset femoral stem (size 3, stainless steel, DePuy Synthes, Leeds, UK). Uncemented THA was performed using a DePuy Corail^®^, standard offset, collarless (size 13, Titanium-64, DePuy Synthes, Leeds, UK). An Articul/Eze femoral head (28 mm diameter) was used on all stems. Stem size and placement were determined, and the implantations were made by an experienced orthopaedic surgeon.

After results were obtained from each THA group, a Vancouver Type B1 PFF for both THAs was carried out on the existing constructs. Fractures were simulated 140 mm below the lesser trochanter, distal to the tip of the stem using an electric saw. This was to minimize interspecimen differences due to plate positioning and fracture reduction. A fifteen-hole broad curved 4.5/5.0 locking compression plate (LCP, DePuy Synthes, Zuchwil, Switzerland) was used (Length, 282 mm; 135 mm at fracture site) for fixation. Fixation proximal to the fracture site was achieved using two eight-hole locking attachment plates (LAP, DePuy Synthes, Solothurn, Switzerland) according to manufactures surgical technique, fixed with a connection screw in holes two and five of the LCP. Two 3.5 mm bicortical self-tapping non-locking screws (42 mm) were used for each LAP. Fixation distal to the fracture site was achieved using four 4.5 mm bicortical self-tapping non-locking screws (42 mm). Periprosthetic implants and screws (LCP and LAP) were manufactured from Titanium–64.

### Loading

The distal 40 mm section of the femur was fixed securely in a cylindrical housing using screws and mounted on a material testing machine (Instron, Massachusetts, USA) at 11° adduction in the frontal plane, and aligned vertically in the sagittal plane to simulate an anatomic one-legged stance [16]. Constructs were tested under displacement control at a rate of 2 mm/min to a maximum of 500 N to avoid damage to the constructs that would affect repeatability [17, 18]. Loading was applied to the head of the femoral stem via a hemispherical cup [18].

### Measurements and analysis

All eight constructs were tested to obtain the overall stiffness of each specimen under the THA and PFF condition. Stiffness was calculated from the slope of the load-displacement data. Fracture movement on the PFF constructs was quantified by measuring the fracture gap of each construct before and after loading on the medial side of the construct. The specimen with the value closest to the overall average stiffness and fracture movement from each group was then used to measure strain.

### Strain gauges

Uniaxial strain gauges were attached at seven sites for the THA group and eight sites for the PFF group. All gauges on the bone and plate were positioned so that the axes were aligned with the longitudinal axis of the femur. Gauges on the stem were aligned with the longitudinal axis of the stem neck. Two gauges with a gauge length of 0.2 mm (FLGB-02-17, Tokyo Sokki Kenkyujo, Tokyo, Japan) were placed on the medial and lateral side of the stem neck (S1–S2), five gauges with a gauge length of 3 mm (GFLAB-3-50, Tokyo Sokki Kenkyujo, Tokyo, Japan) were attached to the surface of the femur for the THA group. Four gauges were positioned on the medial length of the femur at 0, 40, 80, and 200 mm distal to the lesser trochanter, with an additional gauge positioned on the lateral side of the femur, 200 mm distal to the lesser trochanter (B1–B5). Three gauges were positioned on the medial length of the femur at 0, 40, and 80 mm distal to the lesser trochanter for the PFF group (B1–B3). Three strain gauges with a gauge length of 0.2 mm were positioned on the titanium fracture plate around the empty screw hole closest to the fracture gap (P1–P3 – Figure 1B – [17, 18]). It should be noted that strain values obtained are principle strains. Values in positive denote strain in tension, and values in negative denote strain in compression.

### Digital image correlation (DIC)

A stereo DIC system consisting of a pair of two high-resolution cameras was used to create a stereo view of the surface from which the surface strains were calculated using the DIC programme Vic-3D 8 (Correlated Solutions Inc, Irmo, SC, USA). DIC stereo image pairs were recorded from the two cameras and processed using the VIC-3D 8 software. The surface distributions of the maximum principle strains were analyzed. A white-on-black speckle pattern using high contrast spray paint was created on the medial side of the bone for the THA specimens and the area of the fracture site on the fracture plate for PFF specimens [19].

### Statistical analysis

Two-tailed, unpaired Student t-test at a level of significance of *P* < 0.05 was used to detect significant differences in the stiffness, strain, and fracture movement. It should be noted that the strain measurements were performed only on one specimen in each group; hence the *P*-value (for strain measurements) is representative of measurement variability based on the results from the same sample that have been repeated six times.

## Results

**Figure 2:**
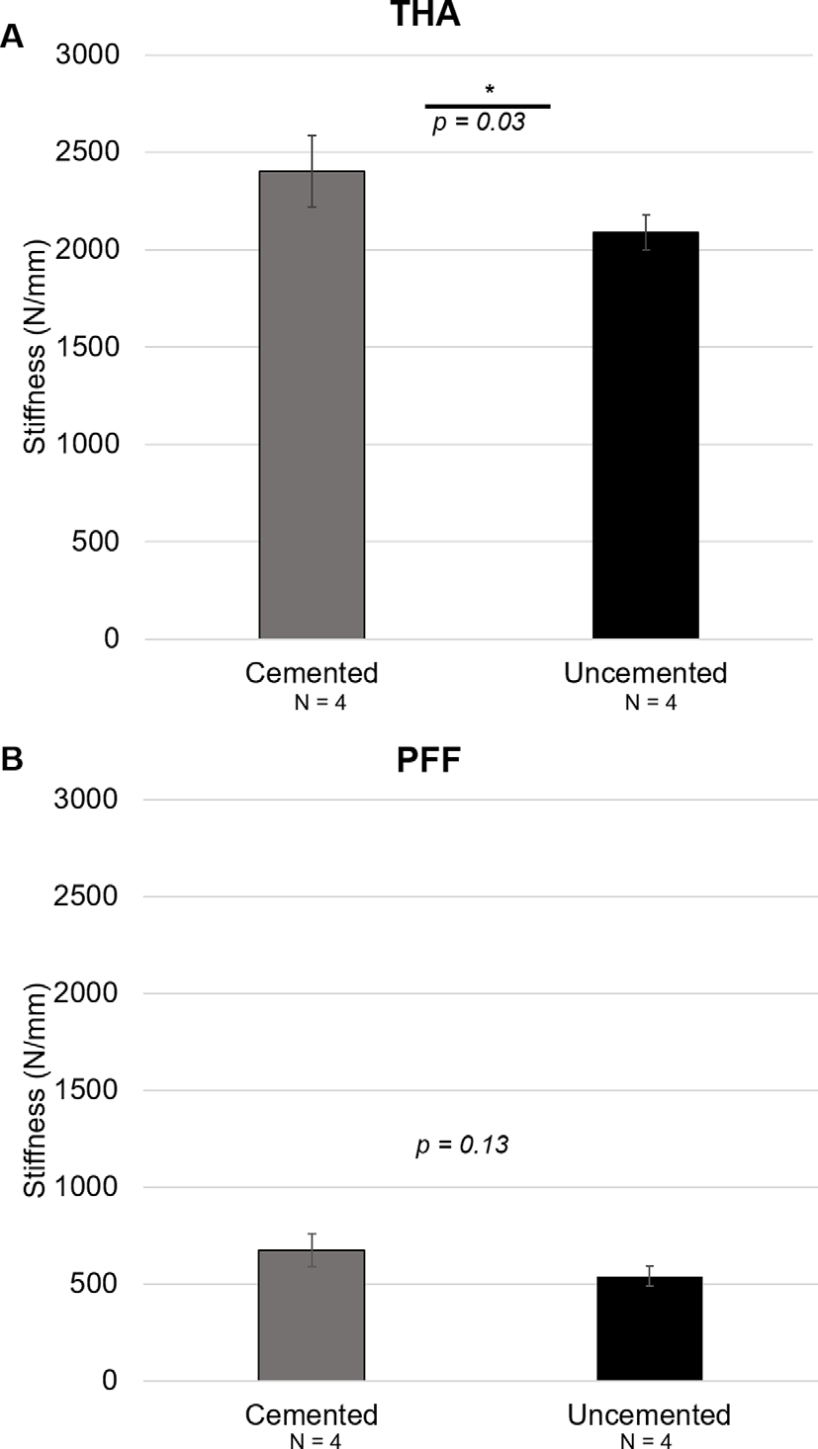
Comparison of stiffness between Cemented and Uncemented specimens based on the overall average in THA group (A) and PFF group (B). *denotes statistical difference between the two variables (*P* < 0.05).

The overall stiffness of the cemented constructs was higher than the uncemented constructs in both the THA and PFF fixation models. A comparison of the average overall stiffness in the THA constructs is shown in Figure 2A. The cemented constructs were significantly stiffer in axial compression than the uncemented ones (*P* = 0.03). A comparison of the average stiffness in the PFF constructs is shown in Figure 2B. Again, the cemented constructs showed a higher overall stiffness than uncemented constructs, albeit this was not statistically significant (*P* = 0.13).

**Figure 3:**
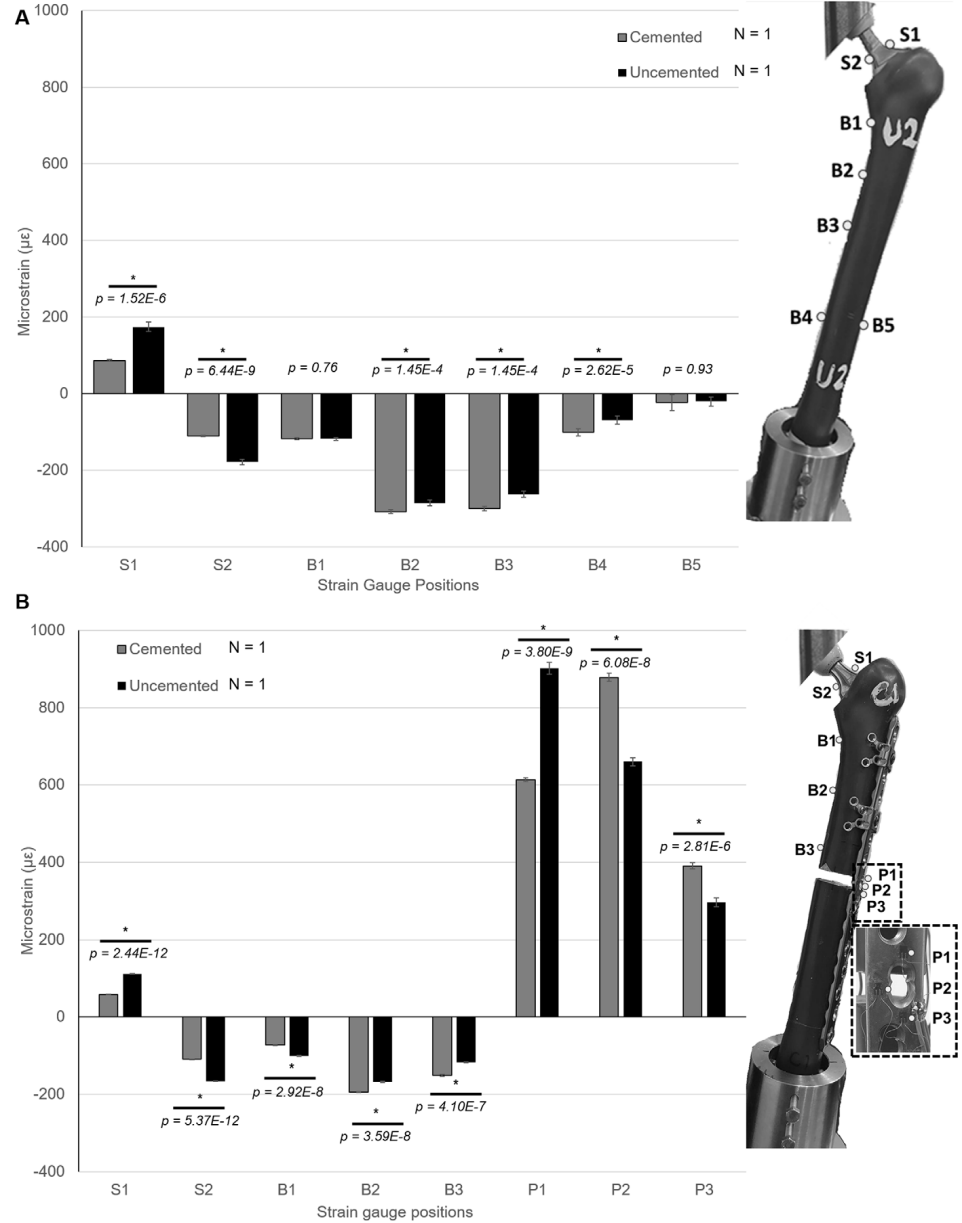
Summary of the strain measurements taken from strain gauges on different locations of the construct under a 500 N axial load in THA cemented vs. uncemented constructs (A) and in cemented and uncemented PFF fixation constructs (B). *Highlights statistical difference between corresponding groups (*P* < 0.05).

Strain gauge results showed that the level of mechanical strain on the uncemented stem was higher than the cemented stem in both the THA and PFF fixation models (see Figure 3A for S1 & S2). There was almost no difference in the strain levels at B1 and B5; however, the cemented construct showed higher strain at B2–B4 (*P* > 0.05). The highest strain for both groups was at B2 (−307.83 ± 4.54 με for the cemented construct and −285.11 ± 7.45 με for the uncemented construct) and B3 (−285.11 ± 7.45 με for the uncemented construct299.89 ± 5.49 με for the cemented, construct and -262.36 ± 7.62 με for the uncemented construct – Figure 3A) and decreasing distally along the bone.

**Figure 4:**
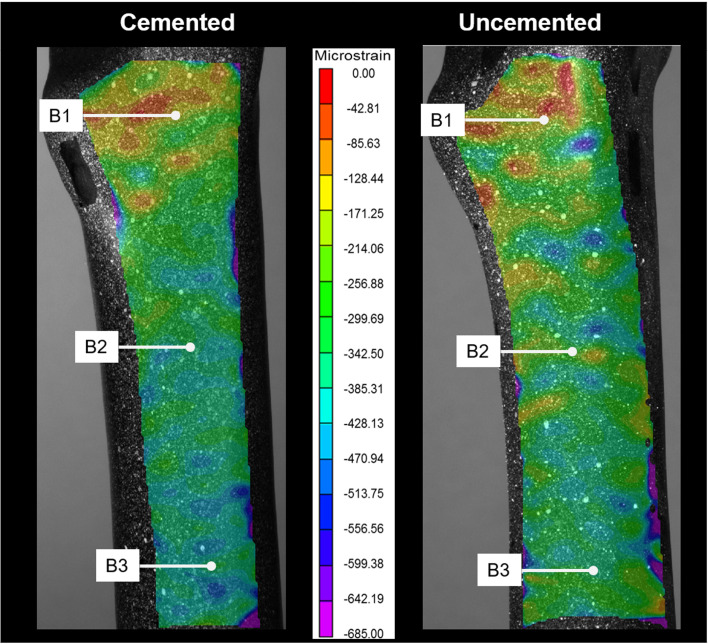
Comparison between the pattern of minimum principal strain across the medial side of the bone, between the uncemented and cemented group at 500 N axial load.

DIC results showed that the overall pattern of the third principle strain (maximum compressive strain) on the medial side of the bone was higher in the cemented construct compared to the uncemented construct, with the exception in B2, where the uncemented construct showed slightly higher strain, this was in good agreement with strain gauge data (Figure 3). Nonetheless, both DIC and strain gauge measurements highlighted that strain was lower at B3 and higher at B1 and B2 (Figure 4).

There was a significant difference in strain gauge data between the cemented and uncemented constructs across all the measurement areas. In both constructs, the highest strain was found on the plate (Figure 3B). Strain across the empty screw hole at the fracture site (P2) was 878.64 ± 10.32 με and 660.21 ± 10.85 με for cemented and uncemented respectively.

Uncemented construct had higher strain at locations P1, S1–S2, and B1, whereas cemented construct had a higher strain at all other locations (Figure 3B). Interestingly, the same strain pattern decreasing distally along the bone was also observed in the PFF constructs. Strain measurement at locations S1–S2 and B1 shows little difference in THA and PFF models.

**Figure 5:**
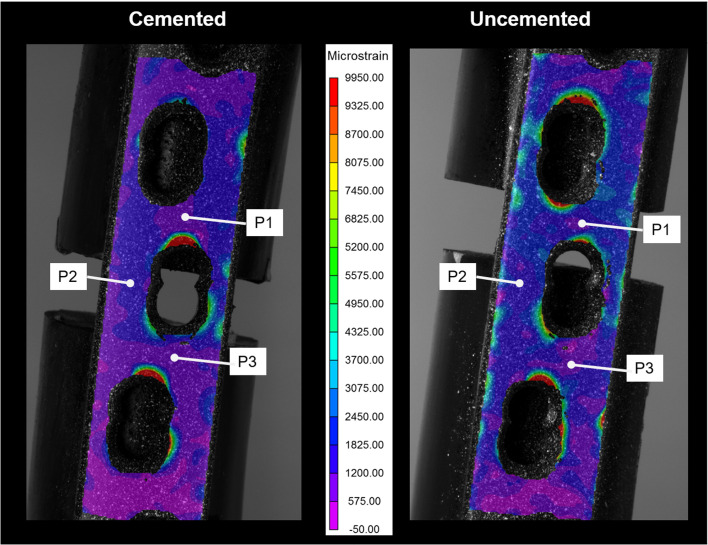
Comparison between the pattern of maximum principal strain across the fracture plate at site of fracture, between the uncemented and cemented group at 500 N axial load.

DIC results show that the overall pattern of the first principal strain (maximum tensile strain) on the lateral side of the fracture plate across the empty screw hole was higher in the uncemented PFF construct compared to the cemented PFF construct (Figure 5). This is contradictory to strain gauge data, where the opposite was found. It must be noted that the DIC data for the uncemented PFF construct was much higher compared to the strain gauge data.

**Figure 6:**
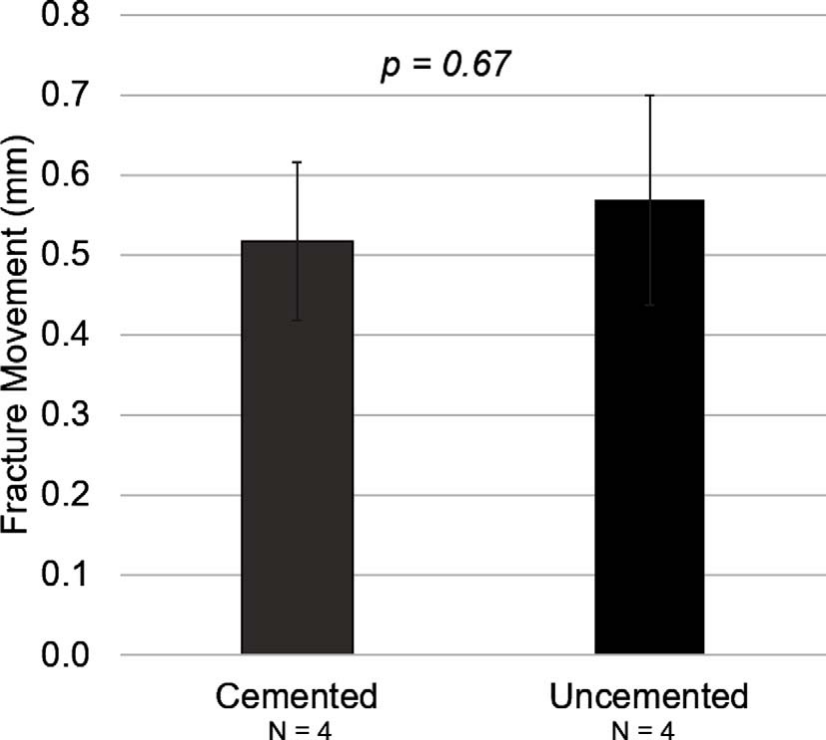
Fracture movement data for cemented and uncemented constructs. Measurements were taken at the fracture site on the medial side of the construct.

Considering all specimens, the overall fracture movement was slightly higher in the uncemented constructs (0.57 ± 0.13 mm) compared to the cemented constructs (0.52 ± 0.10 mm). However, this was not statistically significant (*P* = 0.67 – Figure 6) yet in line with the overall stiffness data.

## Discussion

The biomechanical studies have highlighted that the cemented THAs have a higher overall stiffness compared to the uncemented THAs (hence their use in the elderly population – [8, 9]). However, we do not know if PFFs of well-fixed uncemented THAs have a higher risk of PFF fixation failure compared to their cemented counterparts or not. Results of this study effectively highlight that this is the case, i.e., PFF fixation of uncemented THA considered in this study showed a lower overall stiffness and a higher level of strain on the fracture plate as well as a higher level of fracture movement, in comparison to the PFF fixation of the cemented THA considered in this study.

The major limitation of this study was that (1) C-Stem and Corail stems were used to perform the THAs, while we were conscious of the differences in cross-sectional geometry, size, material, and mode of fixation philosophy between these two implants; nonetheless, this was done so that the experiment could reflect decisions made in the current clinical practice; (2) the strain gauge and DIC tests were only carried out on one specimen while this is not ideal but in line with several previous studies [14, 15]; (3) the present study does not consider the long-term bone ingrowth into the uncemented implants or changes to the bone properties following the THA [20]; (4) this study reflects the mechanical properties and interface interactions of stem-cement/stem-bone in a synthetic sawbone and not a cadaveric femur, in clinical situations bone is likely to be osteopenic. Considering the aforementioned limitations and several others discussed, it is likely that the absolute values reported in this study would be different from the in vivo data. Nonetheless, results provide an understanding of directional suggestions that can be investigated in more clinically relevant study set-ups.

The results show that cemented THA exhibits higher overall stiffness compared to uncemented THA. This is also consistent with the classical application of cemented vs. uncemented implants, where cemented implants are widely used in the elderly population to increase the overall stiffness of what is thought to be a more osteoporotic bone, while uncemented implants are used in younger patients with a higher level of activities and better bone quality. Another factor to consider that could lead to potential differences between the two implantation methods is the difference between the standard offset of C – stem (37.5) and Corail (41.5). The offset of the stem may alter the moment of the arm; however, it would be challenging to characterize the exact contribution of this difference given the number of differences between the constructs considered in this study. Nonetheless, in this respect, the study of Matsushita et al. [21] compared three different femoral offsets in an uncemented implant using a modular neck, they found that increasing the femoral offset from standard (0 mm) to a 4 mm and 8 mm, resulted in 21.1° and 26.7° of improved flexion angle, and 13.7° and 21.2° of improved internal rotation, respectively. Further, Rod Davey et al. [22] suggested that the lever arm of the bending moment increases because of an increased offset, whereas the bending moment only marginally increases due to a decrease in the resultant force. Hence, the net change in strain in the medial cortex is thought to be small.

The major finding of this study was a higher overall pattern of strain on the fracture plate in the uncemented construct compared to the cemented construct (Figure 5). This is important as it suggests that PFF fixation of an uncemented THA can potentially be at a higher risk of failure compared to its cemented counterparts. The present study raises the question that the existing treatment algorithms [6, 7] for B1 PFF management need to consider the cemented vs. uncemented stem implantation method. Various non/clinical parameters should be considered in the clinical decision-making process, such as the bone quality, fracture stability, and the mechanical properties of the implant (Titanium vs. Stainless steel) [17, 23–25]. However, given differences in the design parameters of the cemented and uncemented stems considered in this study, further studies are required to eliminate this variable. In this respect, this study can be regarded as a preliminary investigation for other future studies.

## Conclusions

This study highlights the biomechanical differences between a cemented and uncemented THA when a PFF occurs and is fixed with a plate. It suggests that PFF fixation of a Vancouver type B1 fracture using a plate may have a different risk of failure pending the underlying cemented or uncemented THA implant. However, this requires further investigations to understand the impact of various stem design variables.

## Conflict of interest

The authors declare no conflict of interest.

## Funding

This work was supported by DePuy Synthes (Leeds, UK) and EPSRC Doctoral Training Partnership (DTP) Case Studentship (539270/173067).

## Ethical approval

Ethical approval was not required.

## Informed consent

This article does not contain any studies involving human subjects.

## Authors contributions

KW (First author): performed investigation and acquisition of data, formal analysis, interpretation of data, writing – original draft, review and editing.

MM (Corresponding author): conceptualization, formal analysis, supervision, methodology, project administration, writing – review and editing.

EK, ZG, KS: performed laboratory work and investigation, writing – review and editing.

MA, DH, JT: conceptualization, resources, supervision, writing – review and editing.

MM: conceptualization, supervision, writing – review and editing.

ET: conceptualization, supervision, methodology, writing – review and editing.
